# Individual co-variation between viral RNA load and gene expression reveals novel host factors during early dengue virus infection of the *Aedes aegypti* midgut

**DOI:** 10.1371/journal.pntd.0006152

**Published:** 2017-12-19

**Authors:** Vincent Raquin, Sarah Hélène Merkling, Valérie Gausson, Isabelle Moltini-Conclois, Lionel Frangeul, Hugo Varet, Marie-Agnès Dillies, Maria-Carla Saleh, Louis Lambrechts

**Affiliations:** 1 Insect-Virus Interactions Group, Department of Genomes and Genetics, Institut Pasteur, Paris, France; 2 Centre National de la Recherche Scientifique, Unité de Recherche Associée 3012, Paris, France; 3 Viruses and RNA Interference Unit, Department of Virology, Institut Pasteur, Paris, France; 4 Centre National de la Recherche Scientifique, Unité Mixte de Recherche 3569, Paris, France; 5 Plate-forme Transcriptome & Epigenome, Biomics, Centre d’Innovation et Recherche Technologique (Citech), Institut Pasteur, Paris, France; 6 Hub Bioinformatique & Biostatistique, Centre de Bioinformatique, Biostatistique et Biologie Intégrative (C3BI), Institut Pasteur, Paris, France; 7 Centre National de la Recherche Scientifique, Unité de Services et de Recherches 3756, Institut Pasteur, Paris, France; Johns Hopkins University, Bloomberg School of Public Health, UNITED STATES

## Abstract

Dengue virus (DENV) causes more human infections than any other mosquito-borne virus. The current lack of antiviral strategies has prompted genome-wide screens for host genes that are required for DENV infectivity. Earlier transcriptomic studies that identified DENV host factors in the primary vector *Aedes aegypti* used inbred laboratory colonies and/or pools of mosquitoes that erase individual variation. Here, we performed transcriptome sequencing on individual midguts in a field-derived *Ae*. *aegypti* population to identify new candidate host factors modulating DENV replication. We analyzed the transcriptomic data using an approach that accounts for individual co-variation between viral RNA load and gene expression. This approach generates a prediction about the agonist or antagonist effect of candidate genes on DENV replication based on the sign of the correlation between gene expression and viral RNA load. Using this method, we identified 39 candidate genes that went undetected by conventional pairwise comparison of gene expression levels between DENV-infected midguts and uninfected controls. Only four candidate genes were detected by both methods, emphasizing their complementarity. We demonstrated the value of our approach by functional validation of a candidate agonist gene encoding a sterol regulatory element-binding protein (*SREBP*), which was identified by correlation analysis but not by pairwise comparison. We confirmed that *SREBP* promotes DENV infection in the midgut by RNAi-mediated gene knockdown *in vivo*. We suggest that our approach for transcriptomic analysis can empower genome-wide screens for potential agonist or antagonist factors by leveraging inter-individual variation in gene expression. More generally, this method is applicable to a wide range of phenotypic traits displaying inter-individual variation.

## Author summary

Dengue virus (DENV) is transmitted among humans by mosquitoes, primarily *Aedes aegypti*. Despite their potential as targets to interrupt DENV transmission, mosquito genes that modulate infection in *Ae*. *aegypti* remain largely unknown. Using a field-derived *Ae*. *aegypti* population, we observed substantial variation in DENV load in the mosquito midgut. We hypothesized that this inter-individual variation contained valuable information to identify host factors modulating viral infection. We analyzed single-midgut transcriptomes using an approach that takes advantage of inter-individual variation among infected mosquitoes. We demonstrated the added value of this method by identifying novel host factors during early DENV infection of *Ae*. *aegypti* that went undetected by conventional pairwise comparison between DENV-infected and control groups. We confirmed the agonist role of a candidate gene encoding a sterol regulatory element-binding protein, which underlines the importance of lipid metabolism during DENV infection of the mosquito midgut. Our method for transcriptomic analysis can enhance genome-wide screens for host factors by taking advantage of inter-individual variation. It is also applicable to a wide range of phenotypic traits displaying inter-individual variation.

## Introduction

Dengue virus (DENV) is a mosquito-borne RNA virus of the *Flavivirus* genus (family *Flaviviridae*) that causes an estimated 390 million human infections annually [1]. Although the first dengue vaccine was recently approved in a few countries [2,3], its potential impact is still uncertain [4]. In the absence of specific therapeutics, dengue prevention is limited to vector control, which can be effective but is difficult to sustain in the long term [5].

DENV exists as four serotypes (DENV-1, -2, -3 and -4) that are phylogenetically related and loosely antigenically distinct [6]. DENV has a positive-sense, single-stranded RNA genome that encodes only three structural proteins and seven non-structural proteins. Due to this minimal genetic material, DENV depends on numerous host cellular factors to complete its lifecycle that represent promising targets for the development of antiviral strategies [7,8]. Accordingly, recent genome-wide screens identified multiple human and insect factors required for DENV infectivity [9–12]. For example, several endoplasmic reticulum-associated proteins are necessary for *Flavivirus* infection in both human and insect cells [9,12]. Functional validation *in vivo* in *Aedes* mosquitoes is an important step of such genome-wide screens because candidate host factors identified in model systems are not necessarily confirmed in more biologically relevant organisms. For instance, when the orthologues of three candidate host factors identified in *Drosophila* cells were tested in the main DENV vector *Aedes aegypti*, only one had a conserved function in mosquitoes *in vivo* [11].

One difficulty associated with *in vivo* experiments is that multiple tissues can become infected and may display tissue-specific responses [13,14]. In the field, mosquitoes acquire DENV infection after feeding on a viremic host. Following the infectious blood meal, DENV infection is initially established in the mosquito midgut before the virus spreads systemically to infect the salivary glands and is eventually released in the saliva, through which it is transmitted to the next host [15]. Anatomical barriers to DENV propagation in *Ae*. *aegypti* have been described, namely a midgut infection barrier and a midgut escape barrier [16]. These tissue barriers are quantitative genetic traits controlled by the mosquito genotype [17–19] and specific interactions between mosquito and virus genotypes [20]. Viral genetic determinants [21], the mosquito RNA interference (RNAi) pathway [22,23], and putative receptors [24] have been suggested to mediate these barriers, but overall their molecular nature is still poorly understood [25]. With a few exceptions [26–28], the specific mosquito genes that modulate DENV infection in the midgut of *Ae*. *aegypti* remain to be identified.

Earlier functional genomics studies of DENV infection in the *Ae*. *aegypti* midgut focused on mosquito innate antiviral immunity [26,29–31], or documented transcriptome-wide patterns of gene expression upon DENV exposure [13,14,32,33]. It is worth noting that all these studies used either reference laboratory strains of *Ae*. *aegypti*, such as the Rockefeller and the Liverpool strains, or mosquito lines artificially selected for DENV resistance or susceptibility. Although transcriptomic responses may substantially vary between different *Ae*. *aegypti* strains [13,34], laboratory strains are experimentally powerful because their usually high level of inbreeding minimizes inter-individual variation. To further reduce inter-individual variation, most of these earlier studies examined differential gene expression based on pools of mosquitoes.

Here, we used an alternative functional genomics approach that takes advantage of inter-individual variation in a field-derived *Ae*. *aegypti* population. Using mosquitoes and a DENV isolate originating from Kamphaeng Phet Province in Thailand, we simultaneously examined the transcriptome of 45 individual midguts by RNA sequencing (RNA-Seq) following oral DENV exposure. In addition to a conventional pairwise comparison of DENV-infected versus uninfected control midguts, we examined the correlation between individual midgut viral RNA load and gene expression level among DENV-infected midguts. The aim of the correlation analysis was to identify genes modulating midgut infection without being differentially expressed between DENV-infected and uninfected individuals. For instance, a transcript whose average expression is not significantly different between DENV-infected mosquitoes and uninfected controls would go undetected by pairwise comparison. However, the expression level of this transcript could be significantly correlated with viral RNA load within DENV-infected individuals. Our correlation analysis thus identifies this transcript as a candidate. We demonstrated that this approach has two main advantages. First, it led us to the identification of a set of candidate genes that was not detected by pairwise comparison. Second, the sign of the correlation (*i*.*e*., positive or negative association with viral RNA load) was used to make a prediction about the agonist or antagonist effect of the gene product on virus infection. Agonist refers to a gene promoting virus replication whereas antagonist refers to a gene impairing virus replication. We used Pearson’s determination coefficient as a simple measure of the linear co-variation between viral RNA load and gene expression.

We confirmed the validity of our approach with a candidate gene encoding a sterol regulatory element-binding protein (*SREBP*). SREBPs are transcriptional regulatory proteins conserved among metazoans that modulate lipid biosynthesis [35]. *SREBP* was identified by our correlation analysis but not by conventional pairwise comparison. Positive correlation between *SREBP* expression and DENV RNA load in the midgut was consistent with an agonist effect of this gene. As predicted, *SREBP* knockdown *in vivo* resulted in reduced viral RNA load, revealing a previously unknown agonist role of this mosquito gene during early DENV infection of the *Ae*. *aegypti* midgut.

## Results

### Midgut viral RNA load strongly varies temporally and inter-individually following oral exposure to the same DENV dose

We sampled *Ae*. *aegypti* mosquitoes from a natural population in Thailand and conducted our experiments within the first ten generations of laboratory colonization. In order to preserve its genetic diversity, the colony was maintained as an outbreeding population with several hundreds of reproducing adults at each generation. To examine the temporal dynamics of midgut infection in individual mosquitoes, we monitored DENV genomic RNA concentration in individual midguts of *Ae*. *aegypti* females following exposure to an infectious blood meal containing 1.08 x 10^7^ focus-forming units per mL (FFU/mL) of blood. This infectious dose was chosen to maximize midgut infection prevalence.

**Fig 1:**
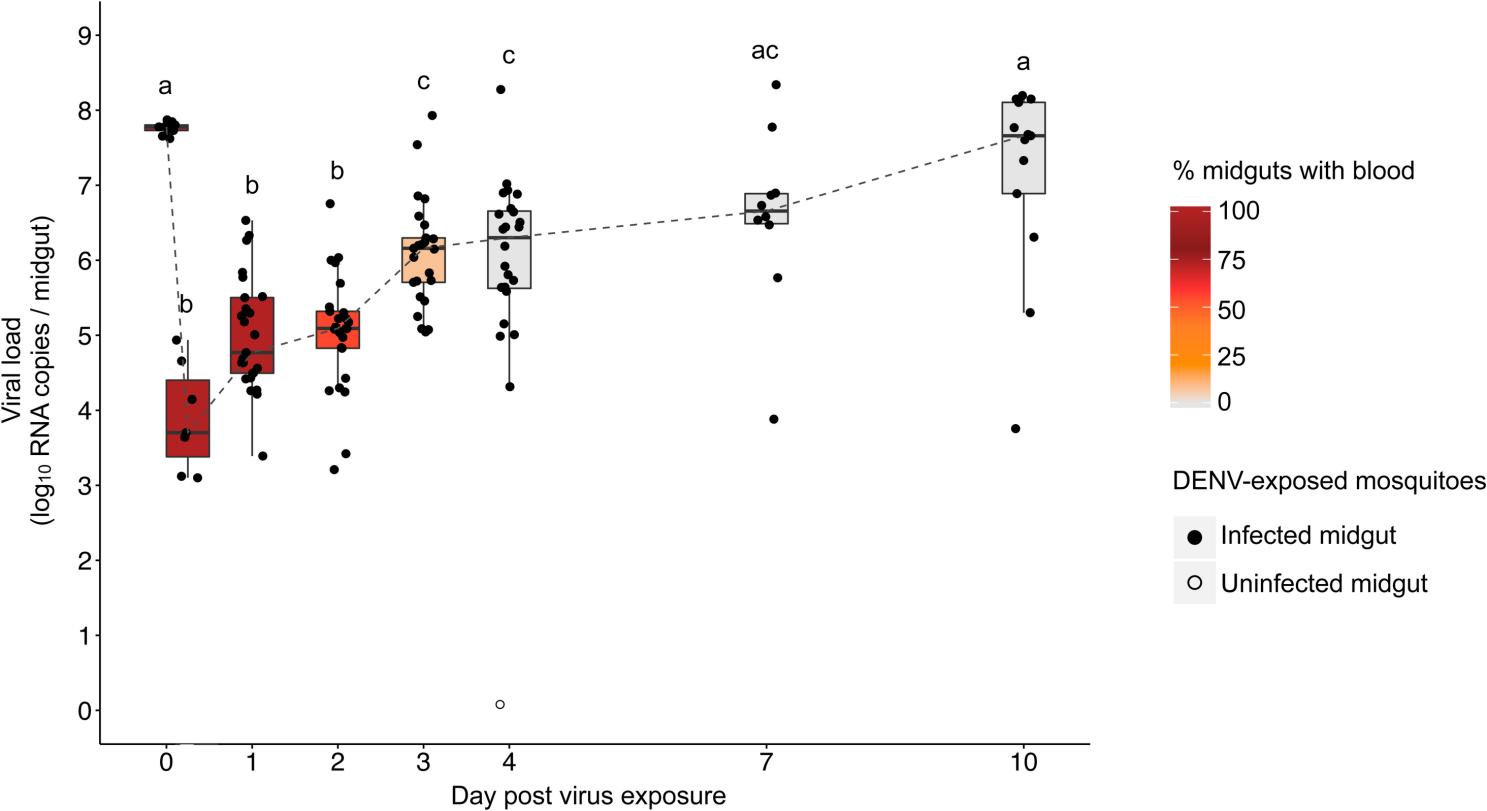
Strong temporal and inter-individual variation in midgut viral RNA load following oral DENV exposure. Time course of viral RNA load measured by RT-qPCR in individual midguts of *Ae*. *aegypti* females following a DENV infectious blood meal. Midguts were harvested immediately after the blood meal (n = 9), at 6 hours (n = 7), 1 day (n = 25), 2 days (n = 25), 3 days (n = 25), 4 days (n = 24), 7 days (n = 10) and 10 days (n = 13) post virus exposure. The color gradient scale indicates the percentage of midguts containing blood at each time point. Solid and open dots represent infected and uninfected midguts, respectively, as determined by RT-qPCR detection threshold. Letters above the graph (a, b, c) indicate statistical differences in the mean viral RNA load between time points according to multiple pairwise t-tests with Holm correction for multiple testing. Viral RNA load is not significantly different (*P* > 0.05) between time points sharing the same letter.

During a 10-day time-course experiment, 137 out of 138 tested midguts were positive for DENV RNA (Fig 1). Based on the presence of undigested blood observed during midgut dissection, blood digestion took up to 4 days (Fig 1). Lack of significant variation in the midgut viral RNA load measured immediately after blood feeding indicated that female *Ae*. *aegypti* ingested similar amounts of DENV (Fig 1, 0 hour post virus exposure). Viral RNA load in the midgut dropped during the first 6 hours post exposure, then increased exponentially for 3 days before reaching a plateau from 7 to 10 days post exposure. Statistical significance of differences across time points is shown in Fig 1. Within each time point, midguts displayed inter-individual variation in viral RNA load as early as 6 hours post exposure. For instance, we observed up to 1,000-fold and 10,000-fold differences in DENV load among individual mosquito midguts on day 1 and day 4, respectively (Fig 1). Viral RNA load can be several orders of magnitude higher than infectious titer [36] but we chose to focus on viral RNA load rather than infectious titers for two reasons. First, we were primarily interested in host factors influencing viral replication and viral RNA load is a better proxy for viral replication efficiency than infectious titer. The latter is a composite phenotype that can be influenced by several other steps than viral replication such as viral particle assembly and maturation. Second, the first few days of mosquito infection by arboviruses are characterized by a so-called eclipse phase during which infectious particles are undetectable [37]. Therefore, our results show that midgut viral RNA load varied significantly not only over the time course but also among individual mosquitoes at a given time point. We next investigated whether this individual variation in viral RNA load could be leveraged to identify novel host factors that modulate midgut infection.

### Pairwise and correlation analyses identify distinct candidate DENV host factors

To identify mosquito genes contributing to natural inter-individual variation in midgut viral RNA load, we used a non-conventional approach for transcriptome analysis. We reasoned that correlating viral RNA load with gene expression at the inter-individual level among DENV-infected mosquitoes could provide information that would be missed by pairwise comparison between DENV-infected and uninfected individuals. To validate our method, we focused on the exponential growth phase of DENV midgut infection (Fig 1). Although successful infection of the midgut is essential for subsequent virus dissemination and transmission, DENV host factors during midgut infection remain largely unknown. Viral dissemination from the midgut to other tissues typically begins around 4 days post exposure [38] and it was confirmed in this mosquito population. We focused on day 1 and day 4 post exposure because they displayed the largest inter-individual variation in viral RNA load (Fig 1). Forty-five individual midguts collected either 1 or 4 days after virus exposure were used for transcriptome analysis by RNA-Seq. They consisted of 16 DENV-infected midguts collected 1 day post exposure, 17 DENV-infected midguts collected 4 days post exposure and 6 control midguts collected at each time point from individuals fed on uninfected blood. The mean number of raw sequencing reads per library that mapped to *Ae*. *aegypti* transcripts was significantly higher on day 1 than on day 4 post exposure (ANOVA: *P* < 0.01), presumably because the digestion process was on-going on day 1 but not on day 4 (S1 Fig). Therefore, we analyzed day 1 and day 4 midgut transcriptomes separately in all subsequent statistical analyses. However, the total number of mapped raw reads per library did not vary significantly between DENV-infected and control midguts (ANOVA: *P* = 0.9).

**Fig 2:**
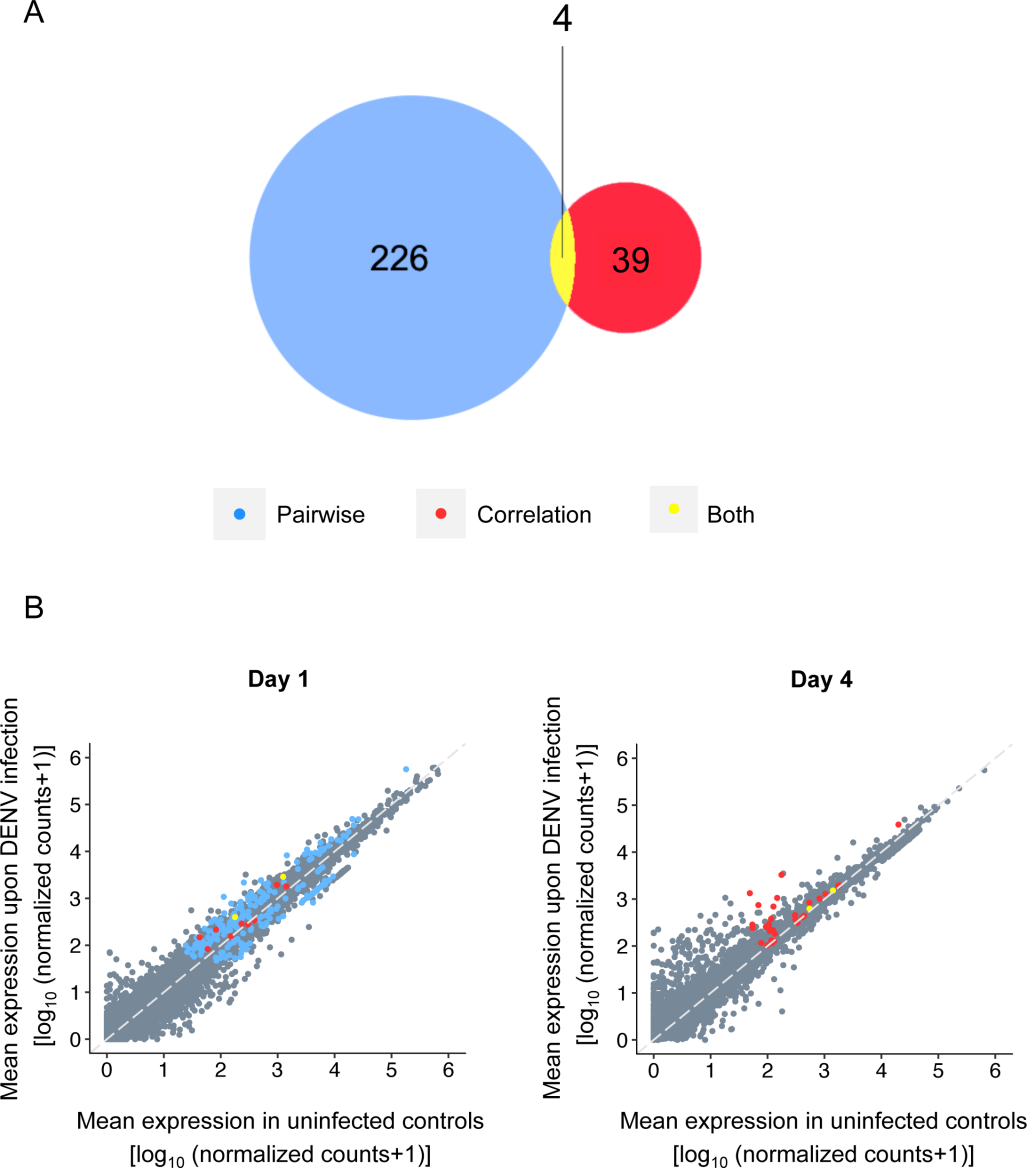
Candidate host factors identified by pairwise comparison and correlation analysis of transcriptome-wide midgut gene expression. (A) Venn diagram showing the number of candidate genes differentially expressed between DENV-infected midguts and uninfected controls (blue) or whose expression was linearly correlated to midgut viral RNA load (red) at a false discovery rate of 0.1, across time points. Common candidate genes between pairwise and correlation analyses are shown (yellow). (B) Time-specific representation of DENV-infected midgut transcriptome plotted against control midgut transcriptome (log_10_ [normalized RNA-Seq counts +1]) at day 1 and day 4 post exposure. Candidate transcripts identified by pairwise comparison (blue), correlation analysis (red) or both (yellow) are indicated.

A total of 13,843 unique mosquito transcripts were detected considering both time points together. To correct for multiple testing, we calculated a false discovery rate (FDR) according to the Benjamini-Hochberg procedure [39]. Based on an FDR threshold of 0.1, we identified 273 *Ae*. *aegypti* candidate transcripts by either pairwise comparison or correlation analysis (S2–S5 Tables). Only four transcripts were detected by both methods across all time points (Fig 2A). By pairwise comparison, 230 transcripts were differentially expressed between DENV-infected and control midguts (Fig 2A). All of these transcripts were identified 1 day post exposure (Fig 2B, blue and yellow dots). The correlation analysis identified 43 candidate transcripts whose expression was correlated with midgut viral RNA load (Fig 2A). The majority of those transcripts were identified 4 days post exposure (Fig 2B, red dots). Among the four transcripts in common between the two methods, two (*AAEL010168* and *AAEL010169)* were both correlated to viral RNA load and differentially expressed at the same time point (day 1) whereas the two others (*AAEL000293* and *AAEL017516)* were detected by the pairwise comparison on day 1 and by the correlation analysis on day 4 (Fig 2B, yellow dots).

According to gene ontology (GO) classification at the biological process level, most of the candidate transcripts belong to metabolism, transcription/translation, oxidation-reduction and proteolysis categories, irrespective of the time point and analysis strategy. Candidates identified by pairwise comparison include transcripts encoding several zinc-finger proteins and immune-related transcripts previously associated with DENV infection in *Ae*. *aegypti* such as the transcription factor *REL1A* [30] and the Complement-related factor *AaMCR* [31] (S2 Table). Several candidates identified by pairwise analysis are genes involved in lipid metabolism, such as the 85-kda calcium-independent phospholipase A2 (*AAEL012835*), a ceramidase (*AAEL007030*), a lipase (*AAEL001837*), a Niemann-Pick-type C2 protein (*AAEL009953*) [27] and a regulator of the Wnt pathway (*AAEL004858*) (S2 Table).

The correlation analysis identified 43 candidate transcripts, of which 39 were not differentially expressed between DENV-infected and uninfected control midguts at any of the time points. The expression level of these genes was linearly associated with midgut viral RNA load, either positively (n = 18) or negatively (n = 21). Because of the statistical association between viral RNA load and gene expression, we hypothesized that the sign of the correlation (*i*.*e*., positive or negative) could predict the effect of the candidate transcript on DENV infection (*i*.*e*., agonist or antagonist). The correlation analysis detected several immune-related genes encoding, for instance, a serine protease inhibitor (*AAEL008364*), a thioester-containing protein 3 (*AAEL008607*) or a leucine-rich immune protein (*AAEL008658*). Expression of immune-related genes was most often negatively correlated with viral RNA load 4 days post virus exposure (S4 Table). Conversely, the expression of two genes involved in lipid homeostasis was positively correlated with viral RNA load 4 days post exposure. One encodes a fatty acid synthase (*AAEL001194*) and the other a sterol regulatory element-binding protein (*SREBP*, *AAEL010555*). To demonstrate the value of our correlation analysis and to provide the proof of concept that the sign of the correlation could be used to make a functional prediction relative to virus infection, we chose gene *AAEL010555* (*SREBP*) for functional validation *in vivo* because of its known role in other viral infections [40–44].

**Fig 3:**
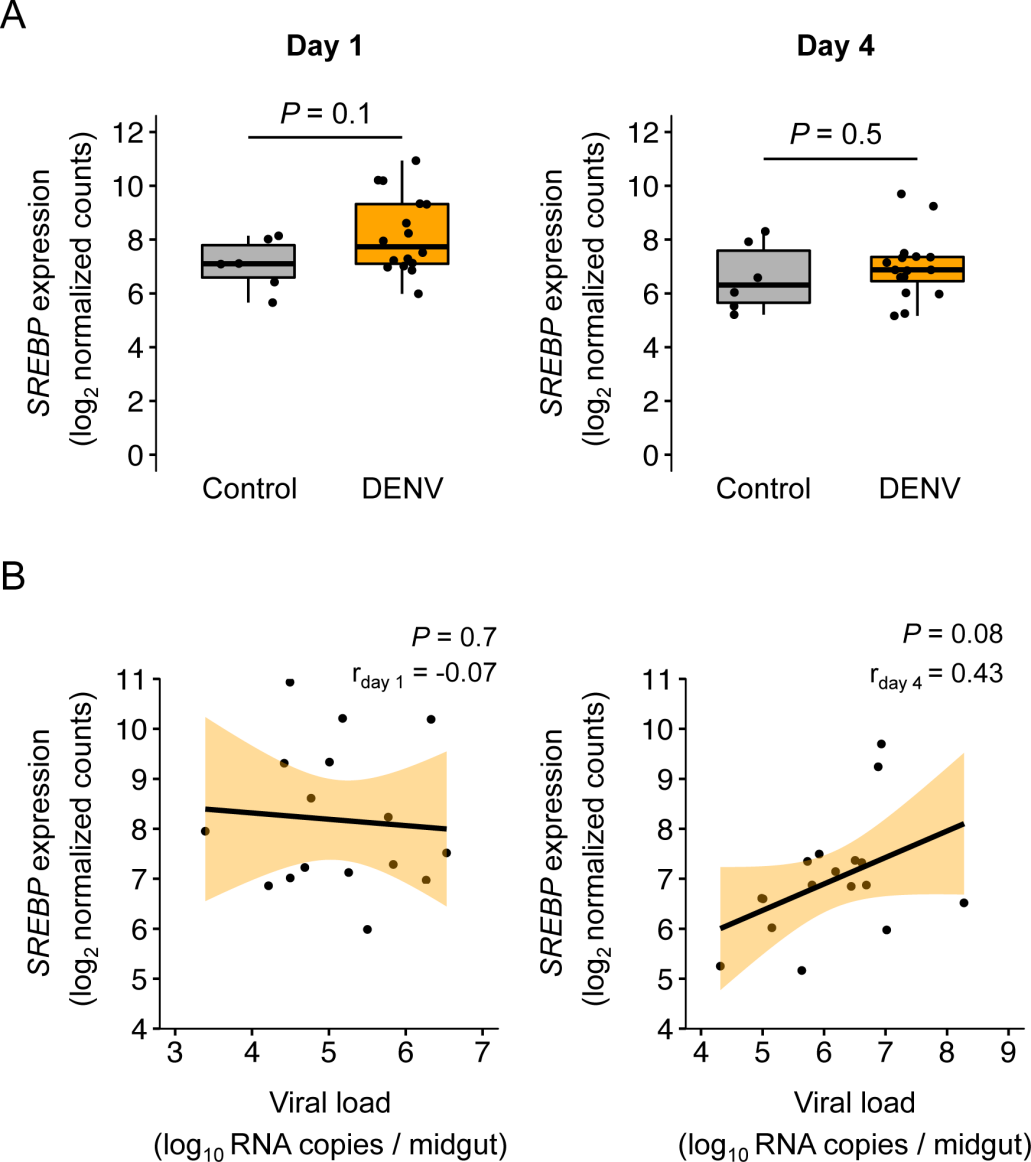
*SREBP* is a candidate host factor during early midgut infection. (A) *SREBP* expression levels measured by RNA-Seq (log_2_-transformed normalized counts) on day 1 and day 4 post DENV exposure in the midgut of control (n = 6) and DENV-infected (n = 16) individuals. *P*-values of the pairwise t-tests are indicated. (B) Correlation of *SREBP* expression level measured by RNA-Seq (log_2_-transformed normalized counts) and viral RNA load (log_10_ RNA copies / midgut) in DENV-infected midguts on day 1 and day 4 post virus exposure. Black lines represent the linear regression and light orange shaded areas represent the 95% confidence intervals. Pearson's coefficients of determination (r) and *P*-values of the linear regression coefficient are indicated.

*SREBP* was not differentially expressed between DENV-infected and control midguts at any of the two time points (Fig 3A). However, midgut viral RNA load and *SREBP* expression were positively correlated on day 4 based on our FDR significance threshold of 0.1 (Fig 3B). The correlation was stronger (r = 0.70; *P* = 0.004) when the three individual midguts with viral loads >10^7^ RNA copies were excluded, consistent with a differential relationship at low versus high viral loads. We predicted that the positive correlation observed for this gene 4 days post exposure indicated an agonist role during midgut infection, and therefore that *SREBP* knockdown during DENV midgut infection would result in reduced viral RNA load.

### *SREBP* knockdown results in ∼50% decrease of midgut viral RNA load

**Fig 4:**
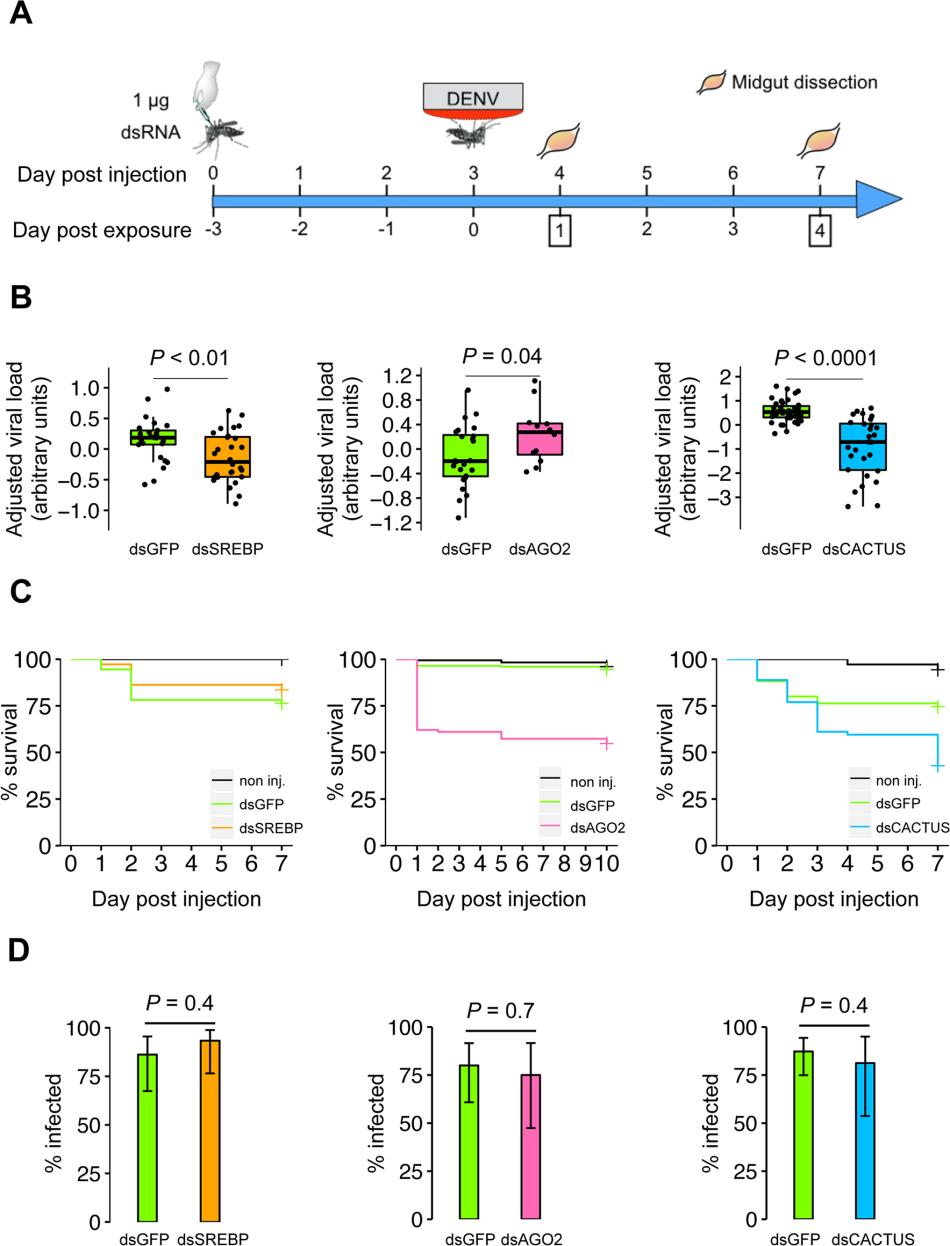
*SREBP* knockdown results in ∼50% reduction of midgut viral RNA load. (A) Time line of gene knockdown assays *in vivo*. (B) Impact of target gene knockdown on midgut viral RNA load. Boxplots represent the viral RNA load measured by RT-qPCR on day 4 post exposure in individual midguts following injection with a single dsRNA. Data represent two (for *SREBP* and *Ago2*) or three (for *Cactus*) separate experiments combined. Viral RNA load was adjusted for differences between experiments and expressed in arbitrary units. On day 4 post exposure, viral RNA load was reduced by 53.8% following *SREBP* knockdown. The *P*-value above the graph indicates statistical significance of the treatment assessed with an ANOVA accounting for the experiment effect. (C) Survival curves following dsRNA injection. Mosquito survival (%) is represented as a function of day post dsRNA injection for individuals injected with dsRNA targeted against *SREBP* (dsSREBP, left), *Ago2* (dsAGO2, center) or *Cactus* (dsCACTUS, right). Mosquitoes injected with dsRNA against GFP (dsGFP) or non injected (non inj.) were used as controls. Each graph of the panel represents a separate experiment. Individuals were exposed to a DENV infectious blood meal 3 days post dsRNA injection. No significant difference in mortality was detected between dsGFP- and dsSREBP-injected mosquitoes according to a Cox model (*P* = 0.3) whereas survival of dsAGO2- and dsCACTUS-injected mosquitoes was impaired relative to the dsGFP controls (Cox model, *P* < 0.0001 for both genes). (D) Effect of target gene knockdown on DENV infection prevalence. Bar plots show the percentage of midguts positive for DENV infection by RT-qPCR on day 4 post virus exposure following injection with a single dsRNA. Data from two (for *SREBP* and *Ago2*) or three (for *Cactus*) separate experiments were combined after verifying the lack of a detectable experiment effect. Vertical bars represent 95% confidence intervals of the percentages. The *P*-value above the graph indicates statistical significance of the treatment effect assessed with a logistic regression.

To test the putative agonist role of *SREBP* during midgut infection by DENV, we used RNAi-mediated gene knockdown assays *in vivo* (Fig 4A). Double-stranded RNA (dsRNA) targeting *SREBP* (dsSREBP) was injected into the thorax of *Ae*. *aegypti* females to reduce *SREBP* expression. Control mosquitoes were injected with the same amount of dsRNA targeting green fluorescent protein (dsGFP). Three days later, we offered mosquitoes a DENV infectious blood meal and quantified viral RNA load in individual midguts by quantitative RT-PCR 1 and 4 days post exposure. *SREBP* knockdown efficiency was 99.7%, 79.3% and 47.0% on day 0, day 4 and day 7 post DENV exposure, respectively (S3A Fig). We observed a significant drop in midgut viral RNA load following *SREBP* knockdown on day 4 post DENV exposure (Fig 4B). There was a 50% reduction in midgut viral RNA load in mosquitoes injected with dsSREBP relative to mosquitoes injected with dsGFP. To further confirm the role of *SREBP* as a DENV agonist, we performed RNAi-mediated gene knockdown assays *in vivo* in a different mosquito population. The field-derived *Ae*. *aegypti* population used for the transcriptomic analysis was originally collected in Thailand. We repeated the experiment in another field-derived mosquito population from Cambodia and also observed a statistically significant reduction of viral RNA load in the midgut following *SREBP* silencing (S2 Fig). In the control groups, the mosquito population from Cambodia had significantly higher prevalence (*P* = 0.0465) but lower viral RNA load (*P* < 0.0001) at day 4 than the population from Thailand. This result is consistent with the agonist role of *SREBP* in DENV replication regardless of the mosquito geographical origin or intrinsic level of susceptibility.

In addition to *SREBP*, RNAi-mediated knockdown was performed against two control genes already known to modulate DENV midgut infection in *Ae*. *aegypti*, *Argonaute-2* (*AAEL017251*, *Ago2*), and *Cactus* (*AAEL000709*) [29,30]. For all target genes (*SREBP*, *Ago2*, *Cactus*), a significant decrease in gene expression was measured on the day of DENV exposure although knockdown efficiency varied between target genes at later time points (S3 Fig). Knockdown of all target genes did not consistently affect the blood-feeding rate, which varied according to the interaction between treatment and experiment (S4 Fig). As expected, *Ago2* knockdown resulted in a statistically significant 42% increase of midgut viral RNA load 4 days post exposure. Note this could be an underestimation, due to the negative feedback loop of using a RNAi-mediated gene silencing assay to knockdown a gene involved in the RNAi pathway. Likewise, *Cactus* knockdown was associated with a statistically significant 81% decrease of DENV load in the midgut 4 days post exposure (Fig 4B). These results were repeatable in at least two separate experiments, thereby validating our gene knockdown assay. Mosquitoes injected with dsRNA against *Ago2* or *Cactus* died faster than dsGFP-injected controls (Cox model: *P* < 0.0001), whereas no significant difference was detected in the survival of mosquitoes injected with dsSREBP or dsGFP until day 7 post injection (Cox model: *P* = 0.3) (Fig 4C). Unlike viral RNA load, the proportion of DENV-infected midguts 4 days post exposure was not influenced by the knockdown of any of the three target genes (Fig 4D).

## Discussion

We used a non-conventional analysis of transcriptomic data to identify new DENV host factors during early midgut infection in a field-derived mosquito population. We observed substantial variation in the individual midgut viral RNA load following oral exposure to the same infectious dose of DENV. This variation presumably results primarily from genetic differences because the viral RNA load measured in midguts on day 0 was almost equal among individuals and all environmental conditions were standardized. Instead of erasing this variation by pooling individual samples prior to transcriptomic analysis, we hypothesized that this variation contained valuable information that could be leveraged. Our approach is expected to identify genes whose expression does not necessarily differ between DENV-infected and uninfected mosquitoes (*i*.*e*., that go undetected by conventional pairwise comparison) but is linearly correlated with viral RNA load. It is worth noting that we used viral RNA load as a proxy for viral replication efficiency in the midgut, which does not directly translate into the level of vector competence. Indeed, viral RNA load in the midgut may not correlate with the probability of virus transmission (e.g., [45]). As a consequence, the candidate genes that we identified do not necessarily meet the requirements to be considered as potential effectors in genetic-based vector control strategies but mainly as host factors agonist or antagonist of viral replication at early time points following infection.

The 230 candidate genes identified by pairwise comparison between DENV-infected and control midguts were detected 1 day post exposure. This indicates that the strongest modulation of midgut gene expression occurs early upon infection. This reinforces observations from previous transcriptomic studies that detected the highest number of differentially expressed genes from 18 to 24 hours post DENV exposure [14,32]. The absence of differentially expressed genes detected 4 days post exposure in our study could result from the use of individual transcriptomes, which reduces the potential bias due to outliers (*i*.*e*., genes with extreme expression levels) that may often exist in mosquito pools. The use of individual midgut transcriptomes allowed identification of 39 genes whose expression was correlated with viral RNA load, in the absence of differential expression between DENV-infected and control individuals. Although this is less than the 226 candidate genes only identified by conventional pairwise comparison, the sign of the correlation allows a strong prediction to be made about the effect of these additional candidates on DENV infection. Only four candidates were detected by both methods, indicating limited overlap between the two analyses and emphasizing their complementarity.

GO classification did not reveal cellular or molecular functions specific to one type of analysis, but numerous genes in the current annotation of the *Ae*. *aegypti* reference genome remain anonymous and lack a predicted function. Improved genome annotations in the future may help to determine whether our correlation analysis and conventional pairwise comparison identify fundamentally different classes of genes. Based on the available annotations, numerous candidate genes were related to lipid metabolism regardless of the analysis. Identification of candidate genes involved in lipid metabolism is consistent with previous studies [13,14,27,46]. To confirm the validity of our correlation approach and to test our hypothesis that the sign of the correlation was predictive of the agonist or antagonist effect of the gene, we focused on a gene encoding a sterol regulatory element-binding protein (*SREBP*). *SREBP* was only detected by the correlation analysis and its expression was positively correlated to viral RNA load, suggesting that this gene promotes virus infection. We confirmed this prediction by RNAi-mediated gene knockdown assays *in vivo*.

*SREBP* genes are conserved among metazoans. Humans harbor three *SREBP* isoforms whereas only one *SREBP* homologue was identified in *Drosophila melanogaster* (*HLH106*) and in *Ae*. *aegypti* [47,48]. SREBPs are membrane-bound transcription factors regulating cholesterol and fatty acid synthesis [49]. In our field-derived *Ae*. *aegypti* population, *SREBP* was not differentially expressed between DENV-infected and uninfected control midguts, in contrast with an earlier study that reported down-regulation of this gene after DENV exposure in pools of mosquitoes from a laboratory strain of *Ae*. *aegypti* [14]. Whether this discrepancy results from differences between mosquito strains, sampling strategy (pooling versus individual transcriptomes), or other differences in the experimental strategy (virus strain, midgut versus whole body, etc.) is unknown. Studies in mice and flies indicate that *SREBP* is likely an essential gene during early development. *SREBP* knockout increased embryonic lethality in mice, and *Drosophila SREBP* mutants died at the larval stage while dietary supplementation with fatty acids rescued mutants to adulthood [35,50,51]. An earlier transcriptomic study in *Ae*. *aegypti* reported that *SREBP* expression was up regulated following blood uptake [52], which is in line with the fact that lipids from the blood meal are required for oocyte maturation [53]. In our two *Ae*. *aegypti* population, *SREBP* knockdown did not significantly impact short-term adult survival. Conversely, we observed a 35% reduction in mosquito survival within 24 hours following *Ago2* knockdown, and a 15% reduction in mosquito survival associated with *Cactus* knockdown following blood feeding. The fitness cost observed in both control treatments could have resulted from immune impairment or from disruption of other processes regulated by the RNAi and Toll pathways.

Our results demonstrated that *SREBP* is an agonist factor during early DENV infection of the *Ae*. *aegypti* midgut. Although the underlying mechanism remains to be elucidated, *SREBP* knockdown was associated with a 53.8% decrease of DENV RNA load in the midgut of our *Ae*. *aegypti* population from Thailand (and 26.9% in the population from Cambodia; S2A Fig). Knocking down *Ago2*, a critical component of the mosquito antiviral response, resulted in a similar effect size (a 42% increase) in midgut viral RNA load. However, the relatively weak correlation between *SREBP* expression and viral RNA load indicates that other host factors determine the efficiency of viral replication. Our finding is consistent with the central role of lipid homeostasis during viral infections. Lipids are required for efficient replication of numerous viruses in mammalian cells including DENV [54,55]. SREBP proteins are transcription factors that regulate a variety of genes involved in lipid synthesis [56]. Hepatitis C virus, a member of the *Flaviviridae* family, increases the amount of lipid droplets through a DDX3X-IKK-α-SREBP pathway that allows assembly of viral particles in human cells [57]. DENV infection also increases the number of lipid droplets in mammalian cells [58] and recently lipid droplets were suggested to play a role during DENV infection in *Ae*. *aegypti* [46]. Human cytomegalovirus, hepatitis B virus and hepatitis C virus have been shown to activate *SREBP*, which can result in an increase in lipid synthesis to promote viral infection [40–44]. In insects, *Drosophila* C virus replication is attenuated in *SREBP* null mutant flies [59]. Thus, our finding that *SREBP* is a host factor promoting DENV infection in *Ae*. *aegypti* adds to the accumulating evidence for a widespread agonist role of this gene during viral infections.

Our results illustrate how transcriptomic data obtained at the individual level can enhance functional genomics studies and improve our understanding of host-pathogen interactions. Based on transcriptome sequencing of individual mosquito midguts, we took advantage of inter-individual variation in gene expression and midgut viral RNA load by using their co-variation as an indication of a functional relationship. The candidate genes that we identified by this method should be useful for other investigators in the field. Identification of DENV host factors *in vivo* paves the way for future mechanistic studies and may ultimately contribute to the development of novel antiviral strategies. More generally, our transcriptomic approach should be of interest in other organisms because it is applicable to virtually any continuous trait with inter-individual variation.

## Methods

### Ethics statement

The Institut Pasteur animal facility has received accreditation from the French Ministry of Agriculture to perform experiments on live animals in compliance with the French and European regulations on care and protection of laboratory animals. This study was approved by the Institutional Animal Care and Use Committee at Institut Pasteur under protocol number 2015–0032.

### Cells and virus

Mosquito cells (*Ae*. *albopictus* C6/36) were maintained in Leibovitz's L-15 medium (Life Technologies) supplemented with 10% foetal bovine serum (FBS, Life Technologies), 1% non-essential amino acids (Life Technologies) and 0.1% Penicillin-Streptomycin (Life Technologies) at 28°C. DENV-1 isolate KDH0030A was originally derived in 2010 from the serum of a dengue patient attending Kamphaeng Phet Provincial Hospital, Thailand [20]. Informed consent of the patient was not necessary because the virus isolated in laboratory cell culture was no longer considered a human sample. DENV-1 isolate was passaged three times in C6/36 cells prior to its use in this study and full-length consensus genome sequence is available from GenBank under accession number HG316482. Virus stock was prepared in C6/36 cells as previously described [60] and a mock-inoculated flask was prepared simultaneously as a negative control. DENV-1 infectious titer was measured in C6/36 cells using a standard focus-forming assay (FFA) as previously described [60].

### Mosquito oral exposure to DENV

Most experiments were carried out with *Aedes aegypti* mosquitoes derived from a wild population originally sampled in 2013 in Thep Na Korn, Thailand and took place within 10 generations of laboratory colonization. One experiment was carried out with *Ae*. *aegypti* mosquitoes derived from a wild population originally sampled in 2015 in Phnom Penh City, Cambodia and took place 8 generations after laboratory colonization. Experimental infections were carried out as previously described [60]. Briefly, four- to seven-day-old females were offered a washed rabbit erythrocyte suspension mixed 2:1 with pre-diluted DENV-1 KDH0030A viral stock and supplemented with 10 mM ATP (Sigma), to reach an expected titer of 10^7^ FFU/mL. A control blood meal was prepared with the supernatant of mock-inoculated C6/36 cells. Mosquitoes were allowed to blood feed for 30 min through a pig-intestine membrane using an artificial feeder (Hemotek Ltd, Blackburn, UK) set at 37°C. Samples of the blood meals were saved and stored at -80°C for further titration. Fully engorged females were incubated at 28°C, 70% relative humidity and under a 12-hour light-dark cycle in 1-pint cardboard cups (20–30 females per cup, at least 2 cups/condition) with permanent access to 10% sucrose.

### Midgut dissection and RNA isolation

Upon harvest, females were freeze-killed at -80°C and transferred on ice. Midguts were dissected in 1X phosphate-buffered saline (PBS) under 10X magnification. Forceps were decontaminated between each individual using Surfa’Safe (Anios) to prevent cross contamination. Individual midguts were immediately homogenized for 30 sec at 6,000 rpm in tubes (VWR) containing ~20 1-mm glass beads (BioSpec) in 800 μL of TRIzol (Life Technologies) and stored at -80°C. Samples were thawed at room temperature (20–25°C) and 150 μL of chloroform (Sigma-Aldrich) were added followed by vortexing for 30 sec. After a 5-min incubation at 4°C, samples were centrifuged at 4°C for 15 min at 14,000 rpm. The upper aqueous phase was harvested and transferred to a cold tube containing 400 μL of 2-propanol (Sigma-Aldrich) supplemented with 1 μL GlycoBLUE (Ambion, Life Technologies). Samples were incubated at -20°C overnight and centrifuged at 4°C for 15 min at 14,000 rpm to pellet RNA. The pellet was washed with 800 μL of 70% ice-cold ethanol (Sigma-Aldrich) at 4°C for 10 min at 14,000 rpm, and allowed to dry for 10 min at 37°C. Total RNA was resuspended in 6 μL, of which 1 μl was diluted into 9 μl of RNase-free water for DENV quantification by RT-qPCR, while the remaining 5 μl were used for transcriptome sequencing. All the samples were stored at -80°C until use.

### DENV quantification by RT-qPCR

DENV RNA was quantified using *NS5*-specific primers and TaqMan probe (S1 Table) with SuperScript III Platinum One-Step RT-qPCR kit (Life Technologies) and serial dilutions of total DENV RNA of known concentration (from 10^9^ to 10^1^ DENV RNA copies/μL) as a standard, as previously detailed [60]. Each RT-qPCR plate included negative controls derived from uninfected samples and a no template control. The RT-qPCR results were validated if the slope of the standard curve was between -3.33 and -3.65, corresponding to 90–100% efficiency.

### Midgut transcriptome profiling by RNA-Seq

Individual midgut libraries were prepared from total RNA extracts from individual midguts after quality control with a Bioanalyzer RNA 6000 kit (Agilent). Purification and fragmentation of mRNA, cDNA synthesis, end-repair, A-tailing, Illumina indexes ligation and PCR amplification were performed using TruSeq RNA Sample Prep v2 (Illumina) followed by cDNA quality check by Bioanalyzer DNA 1000 kit (Agilent). Libraries were diluted to 10 pM after Qubit quantification (ThermoFisher), loaded onto a flow cell, clustered with cBOT (Illumina). Single-end reads of 51 nucleotides in length were generated on a HiSeq2000 sequencing platform (Illumina). Sequencing reads with a quality score <30 were trimmed using Cutadapt [61]. Passing-filter reads were mapped to *Ae*. *aegypti* transcripts (AaegL3.1, http://vectorbase.org) using Bowtie2 [59] with the “sensitive” option. They were processed with the Samtools suite [62] to create of a matrix of raw counts used for gene expression analysis. The RNA-Seq data were deposited to SRA under accession number PRJNA386455 (https://www.ncbi.nlm.nih.gov/bioproject/386455).

### RNA-Seq statistical analyses

All analyses of midgut transcript expression were performed in R (v. 3.2.3, http://www.r-project.org/) using the DESeq2 package v.1.8.0 [63]. Following normalization of raw read counts by the relative log expression method implemented in DESeq2 [64], normalized read counts were considered separately according to time post DENV exposure. Two complementary analyses were run for each time point. First, a pairwise comparison was used to identify genes differentially expressed between DENV and control conditions. Differential expression was evaluated using the DESeq2 generalized linear model with its default parameters (activated outlier detection and independent filtering). Statistical significance of differential expression was determined based on a 10% false discovery rate (FDR). Second, a correlation analysis in DESeq2 measured the strength of the linear relationship between log_2_-transformed normalized read counts and the log_10_-transformed viral RNA load per midgut in DENV-1 samples only. Statistical significance of the linear relationship was determined based on a 10% FDR threshold. Genes with no detectable or very low expression (*i*.*e*., median < 50 normalized read counts) were filtered out after the statistical analysis.

### Design and synthesis of dsRNA

DNase-treated RNA purified from a pool of *Ae*. *aegypti* midguts was used to produce a PCR template for dsRNA synthesis. Briefly, gene-specific PCR primers for dsRNA preparation were designed (S1 Table) using E-RNAi web-service v.3.2 [65] with 21-bp length for siRNA specificity prediction and default parameters otherwise. A 500-bp fragment of the *GFP* gene was amplified with specific primers (S1 Table) and cloned into pCRII TOPO vector (Life Technologies). A T7 promoter was incorporated into the PCR amplicon with tagged primers (S1 Table). PCR was conducted in a 25-μL reaction containing 2 μL of template cDNA, 5 μM of each T7 primer, 1.5 mM MgCl2, 200 μM of dNTP mix and 0.5 unit of native Taq polymerase (ThermoFisher) as follows: 3 min at 95°C, 40 cycles of 1 min at 94°C, 1 min at 58°C, 1 min at 72°C, and a final step of 10 min at 72°C. Synthesis of dsRNA was performed overnight at 37°C using MEGAscript RNAi kit (Life Technologies) with 1 μg of PCR product purified by MinElute Kit (Qiagen). After column purification, 1:10 (vol/vol) 3M sodium acetate pH 5.5 (Life Technologies) and 1:2.5 (vol/vol) 100% ethanol (Sigma-Aldrich) were added, followed by overnight precipitation at -80°C. After centrifugation for 30 min at 14,000 rpm, the dsRNA pellet was washed with 800 μL of 100% ethanol, followed by 15 min centrifugation at 14,000 rpm. The dsRNA pellet was air-dried, resuspended in RNase-free water, adjusted to a concentration of 7 μg/μL with a Nanodrop spectrophotometer, and stored at -20°C until use.

### Gene knockdown assays *in vivo*

Four- to 7-day-old females were ice-chilled and intrathoracically injected with 2 x 69 nL of a 7 μg/μL dsRNA (~1 μg dsRNA) from the gene of interest using a Nanoject-II device (Drummond). Control mosquitoes were injected with dsGFP. Mosquitoes were allowed to recover from injection for 2 days before being offered an artificial DENV-1 blood meal as described above. Both dsCACTUS and dsAGO2 were used as controls for DENV-1 load modulation in the midgut.

### Gene expression quantification by RT-qPCR

Total RNA from individual midguts was reverse transcribed into cDNA in a reaction mixture containing 5 nM random hexamers, 0.2 mM of dNTP mix, 10 μL of template and RNase-free water up to 14.5 μL. After incubation at 65°C for 10 min, samples were chilled on ice for 5 min. For each reaction, 4 μL of 5X First-Strand buffer, 1 μL of 0.1 mM Dithiothreitol, 40 units of RNase-OUT and 100 units of MML-V reverse transcriptase (Life Technologies) were added to a final volume of 20 μL. After 10 min at 25°C, cDNA synthesis was conducted at 37°C for 50 min and terminated at 70°C for 15 min. cDNA samples were stored at -20°C until use. Gene expression was assayed by relative quantitative PCR (qPCR) using a LightCycler96 machine (Roche). The qPCR mix contained 200 nM of each primer, 10 μL of 2X SYBR-green I Master Mix (Roche) and PCR grade water to 18 μL, with 2 μL of cDNA template to a final volume of 20 μL. Settings were an initial denaturation step of 5 min at 95°C, followed by 40 cycles of 10 sec at 95°C, 20 sec at 60°C and 10 sec at 72°C. Melting curve were used to confirm the absence of non-specific PCR amplicons using the following program: 5 sec at 95°C, 60 sec at 65°C and continuous fluorescence acquisition up to 97°C with a ramp rate 0.2°C/sec. Relative expression was calculated as 2 ^-(Cq^_*gene*_^-Cq^_*rp49*_^)^, using the *Ae*. *aegypti* ribosomal protein-coding gene *rp49* (*AAEL003396*) for normalization.

### Statistical analyses

Infection prevalence was analyzed as a binary response variable (0 = absence, 1 = presence) using logistic regression. Continuous response variables were analyzed using analysis of variance (ANOVA). Explanatory variables included time point (ordinal), experimental condition (nominal) and experiment (nominal). Viral RNA load was log_10_-transformed and RNA-Seq normalized read counts were log_2_-transformed prior to analysis. Midgut gene expression normalized by *rp49* (referred to as expression) was analyzed without log-transformation. Models including interactions were analyzed with type-III ANOVA, whereas models without interactions were analyzed with type-II ANOVA. Interactions terms were removed from the final model if they were not statistically significant (*P* > 0.05). When the ANOVA assumption of normal error distribution could not be met, a non-parametric Wilcoxon test was performed for pairwise comparisons. Multiple pairwise comparisons were performed with t-tests followed by Holm correction for multiple testing [66]. A Cox regression model including dsRNA injection and DENV exposure as covariates was used to compare mosquito survival across treatments [67]. This model is appropriate to analyze the effect of several variables on the time it takes for an event to happen. Statistical analyses were computed in the R environment and plotted with the R package *ggplot2* (v. 2.2.0) [68].

## Supporting information

S1 Fig Size distribution of RNA-Seq libraries prepared from individual midguts. Bars show the total read count in each library prepared from individual mosquito midguts 1 and 4 days after DENV or mock infection. Raw read counts were significantly higher on day 1 than on day 4 (ANOVA: *P* < 0.01) but did not differ between experimental treatments (ANOVA: *P* = 0.9).
(PDF)

S2 Fig *SREBP* knockdown reduces midgut viral RNA load in a field-derived *Ae*. *aegypti* population from Cambodia. (A) Impact of *SREBP* knockdown on midgut viral RNA load. Boxplots represent the log_10_-transformed viral RNA load on day 4 post exposure in individual midguts. The *P*-value above the graph indicates statistical significance of the treatment assessed with an ANOVA. On day 4 post exposure, viral RNA load was reduced by 26.9% following *SREBP* knockdown. (B) *SREBP* expression knockdown in the midgut. Boxplots represent the *SREBP* expression normalized by *rp49* in the midgut of individuals injected with dsSREBP at two time points post DENV exposure. The percentage indicates silencing efficiency. Mosquitoes injected with dsGFP were used as controls. *P*-values above the graph indicate statistical significance of pairwise differences between treatments according to a Wilcoxon test.
(TIF)

S3 Fig Time course of target gene knockdown in individual mosquito midguts. Boxplots represent the target gene expression normalized by *rp49* in the midgut of individuals injected with dsSREBP (A), dsAGO2 (B) or dsCACTUS (C) at different time points post exposure to DENV for one experiment. Percentages indicate silencing efficiency. Mosquitoes injected with dsGFP were used as controls. *P*-values above the graph indicate statistical significance of pairwise differences between treatments according to a Wilcoxon test.
(TIF)

S4 Fig Blood-feeding rates of dsRNA-injected mosquitoes. Barplots show the percentage of blood-engorged females previously injected with dsSREBP (A), dsAGO2 (B) and dsCACTUS (C). Non-injected individuals or individuals injected with dsRNA against GFP were used as controls. The *x*-axis indicates each separate experiment. Vertical bars represent 95% confidence intervals of the percentages. The percentage of blood-fed mosquitoes was significantly influenced by a condition x experiment interaction for *SREBP* (logistic regression: *P* = 0.02), *Ago2* (logistic regression: *P* = 0.02) and *Cactus* (logistic regression: *P* < 0.01) knockdown assays.
(PDF)

S1 Table Primers used for qPCR and dsRNA synthesis. The table provides nucleotide sequences of forward primer (-F), reverse primer (-R) and TaqMan probe (-Probe) for target genes shown in italics. Positions in the target gene (in nucleotide) and amplicon size (in base pairs) are indicated. Primers used for dsRNA synthesis (ds) include a T7 promoter sequence at their 5' end (bold font).
(XLS)

S2 Table Midgut candidate genes identified by pairwise comparison on day 1 post DENV exposure. (XLSX)

S3 Table Midgut candidate genes identified by correlation analysis on day 1 post DENV exposure. (XLSX)

S4 Table Midgut candidate genes identified by correlation analysis on day 4 post DENV exposure. (XLSX)

S5 Table Summary of midgut candidate genes analyzed. The table shows the 7,637 mosquito transcripts detected in the midgut at both day 1 and day 4 post exposure to DENV, after filtering for a median count > 50. Candidate transcripts identified by pairwise analysis (S2 Table) are highlighted in blue and candidate transcripts identified by correlation analysis (S3 and S4 Tables) are highlighted in red, according to the color code in Fig 2.
(XLS)
