# Supplementary figures and images for: Individual co-variation between viral RNA load and gene expression reveals novel host factors during early dengue virus infection of the Aedes aegypti midgut
Source: PLoS Negl Trop Dis. 2017 Dec 19;11(12):e0006152. doi: 10.1371/journal.pntd.0006152 (PMC5752042; doi:10.1371/journal.pntd.0006152)

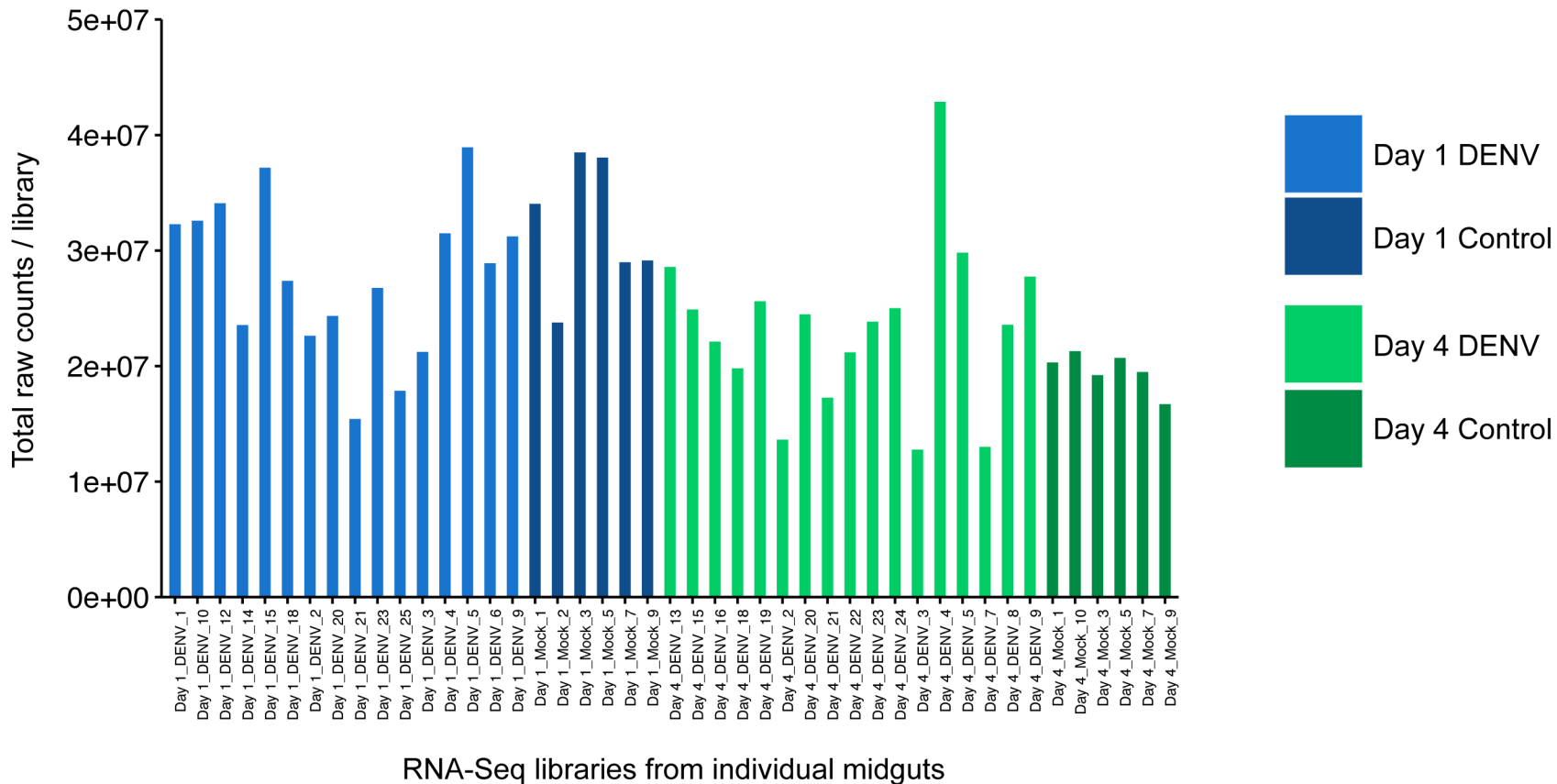

Supplement: S1 Fig — Bars show the total read count in each library prepared from individual mosquito midguts 1 and 4 days after DENV or mock infection. Raw read counts were significantly higher on day 1 than on day 4 (ANOVA: P < 0.01) but did not differ between experimental treatments (ANOVA: P = 0.9). (PDF) [file pntd.0006152.s001.pdf]

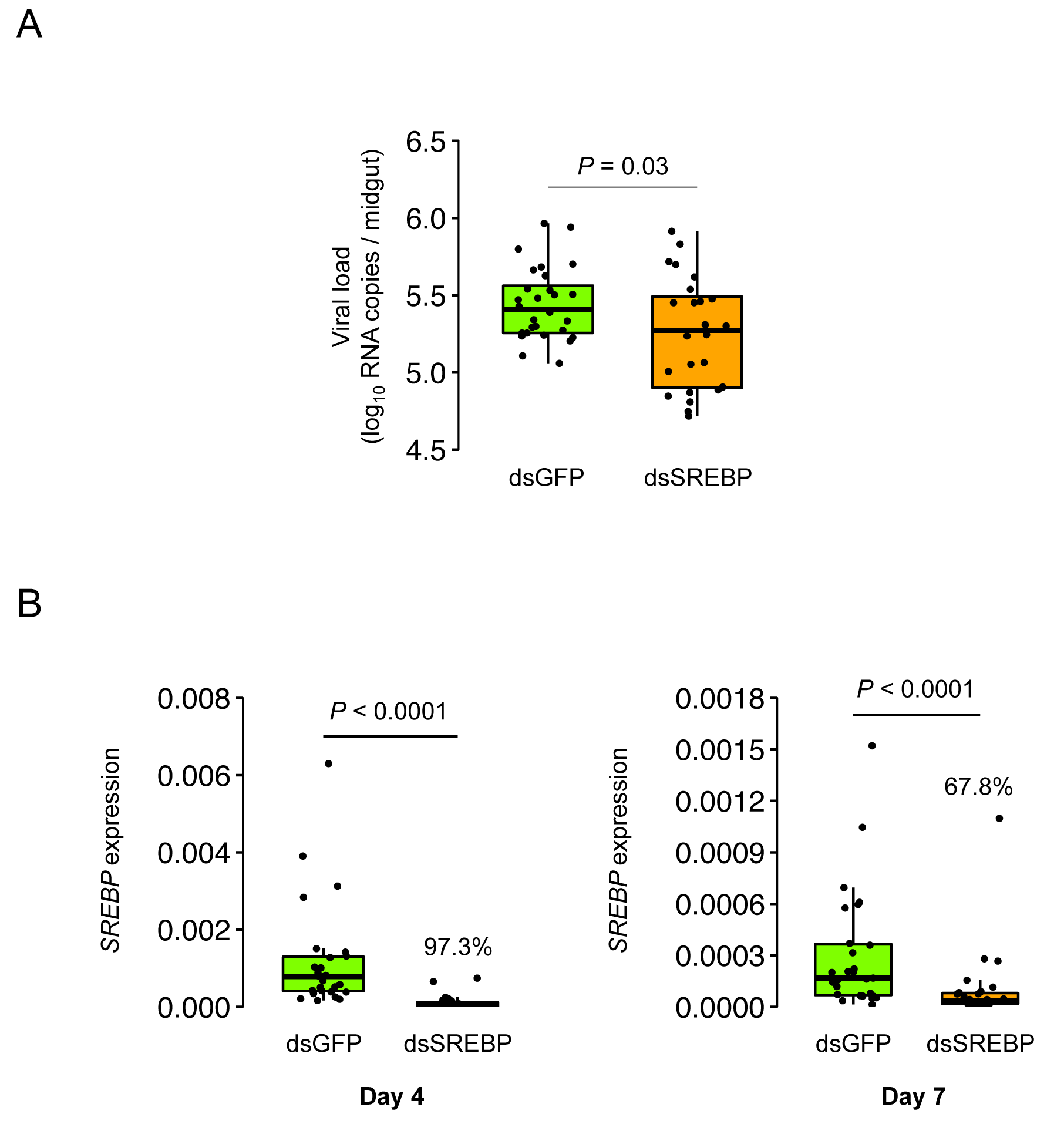

Supplement: S2 Fig — (A) Impact of SREBP knockdown on midgut viral RNA load. Boxplots represent the log10-transformed viral RNA load on day 4 post exposure in individual midguts. The P-value above the graph indicates statistical significance of the treatment assessed with an ANOVA. On day 4 post exposure, viral RNA load was reduced by 26.9% following SREBP knockdown. (B) SREBP expression knockdown in the midgut. Boxplots represent the SREBP expression normalized by rp49 in the midgut of individuals injected with dsSREBP at two time points post DENV exposure. The percentage indicates silencing efficiency. Mosquitoes injected with dsGFP were used as controls. P-values above the graph indicate statistical significance of pairwise differences between treatments according to a Wilcoxon test. (TIF) [file pntd.0006152.s002.tif]

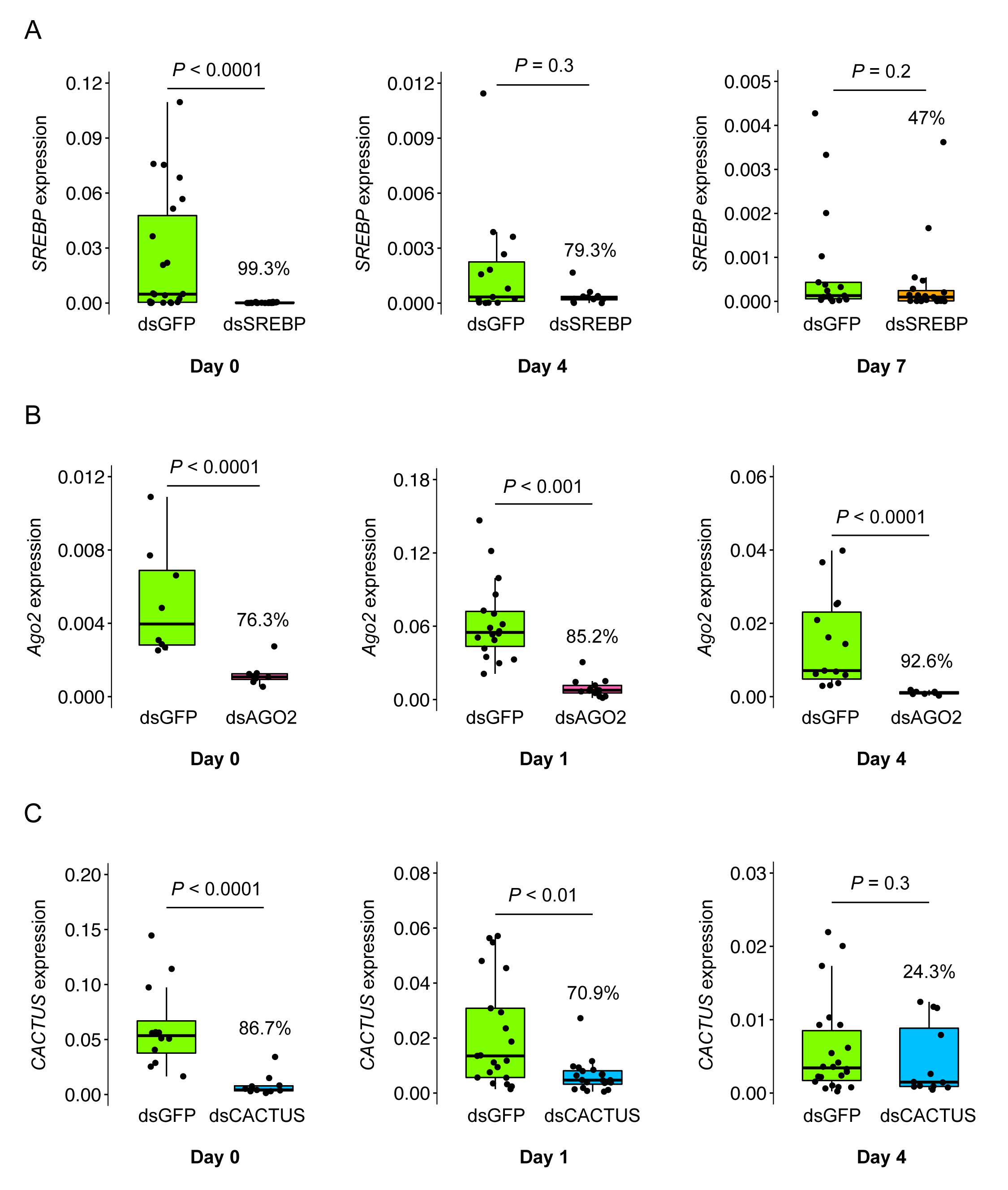

Supplement: S3 Fig — Boxplots represent the target gene expression normalized by rp49 in the midgut of individuals injected with dsSREBP (A), dsAGO2 (B) or dsCACTUS (C) at different time points post exposure to DENV for one experiment. Percentages indicate silencing efficiency. Mosquitoes injected with dsGFP were used as controls. P-values above the graph indicate statistical significance of pairwise differences between treatments according to a Wilcoxon test. (TIF) [file pntd.0006152.s003.tif]

A

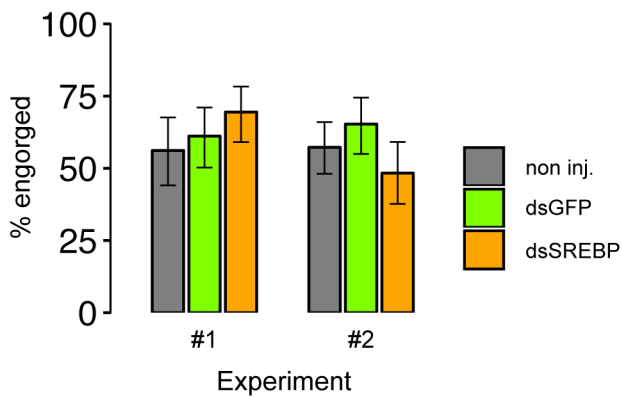

B

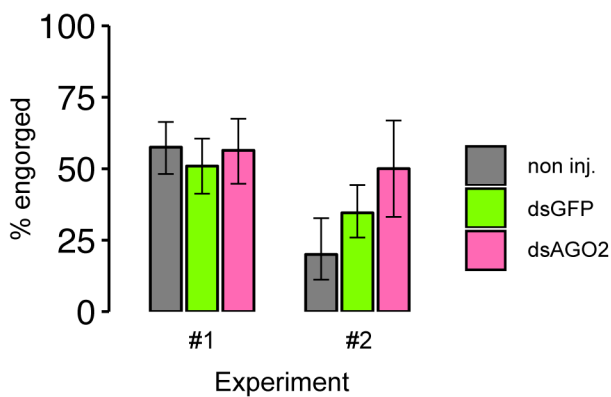

C

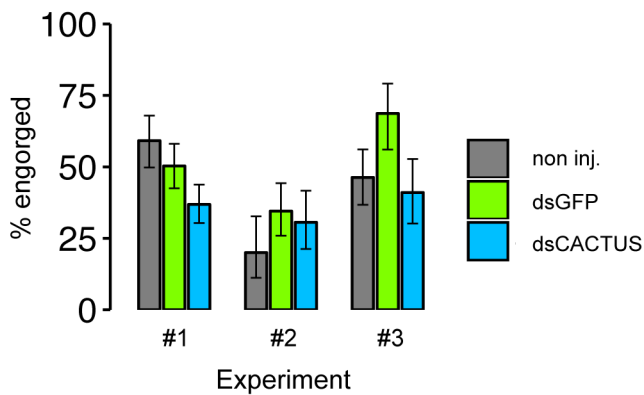

Supplement: S4 Fig — Barplots show the percentage of blood-engorged females previously injected with dsSREBP (A), dsAGO2 (B) and dsCACTUS (C). Non-injected individuals or individuals injected with dsRNA against GFP were used as controls. The x-axis indicates each separate experiment. Vertical bars represent 95% confidence intervals of the percentages. The percentage of blood-fed mosquitoes was significantly influenced by a condition x experiment interaction for SREBP (logistic regression: P = 0.02), Ago2 (logistic regression: P = 0.02) and Cactus (logistic regression: P < 0.01) knockdown assays. (PDF) [file pntd.0006152.s004.pdf]
